# Chemical shift assignment of the ataxin-1 AXH domain in complex with a CIC ligand peptide

**DOI:** 10.1007/s12104-013-9509-z

**Published:** 2013-07-14

**Authors:** Cesira de Chiara, Geoff Kelly, Rajesh P. Menon, John McCormick, Annalisa Pastore

**Affiliations:** 1 MRC National Institute for Medical Research, The Ridgeway, London, NW7 1AA UK; 2 MRC Biomedical NMR Centre, The Ridgeway, London, NW7 1AA UK

**Keywords:** AXH, SCA1, Poly-glutamine, Conformational switch, CIC, Transcriptional repressor

## Abstract

Ataxin-1 is the protein responsible for the genetically-inherited neurodegenerative disease spinocerebellar ataxia type-1 linked to the expansion of a polyglutamine tract within the protein sequence. The AXH domain of ataxin-1 is essential for the protein to function as a transcriptional co-repressor and mediates the majority of the interactions of ataxin-1 with cellular partners, mainly transcriptional regulators. One of the best characterized ataxin-1 functional partners is Capicua (CIC), a transcriptional repressor involved in signalling pathways that regulate mammalian development, tumorigenesis and, through the interaction with ataxin-1, also neurodegeneration. Complex formation of ataxin-1 with CIC is important both for the function of the wild-type protein and for pathogenesis as transcriptional disregulation is observed since the early stages of the development of the disease. Here we report the ^1^H, ^13^C and ^15^N backbone and side-chain chemical shift assignments of the human ataxin-1 AXH domain in complex with a CIC ligand-peptide.

## Biological context

**Fig. 1:**
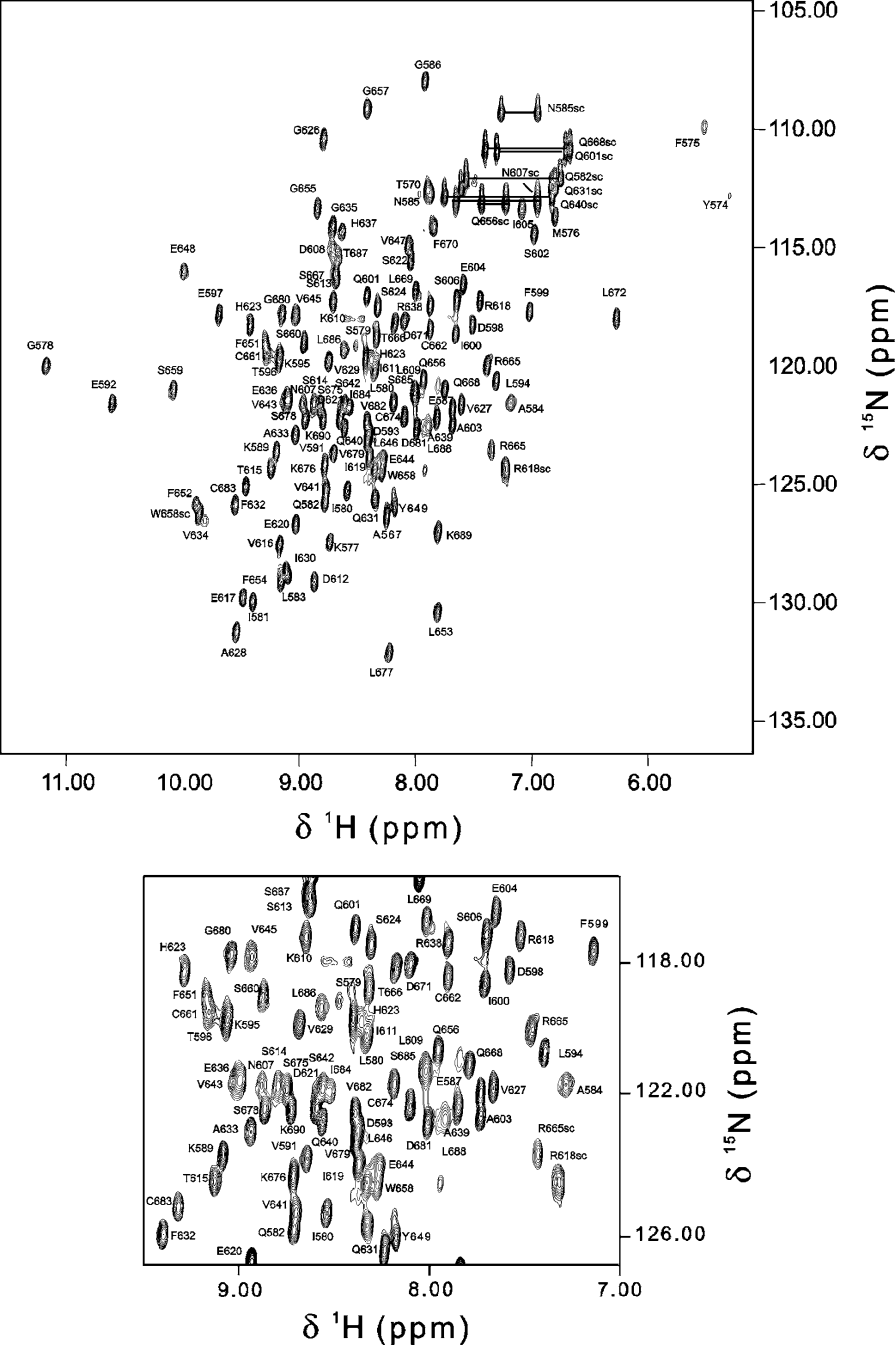
2D ^1^H, ^15^N-HSQC spectrum of ataxin1 AXH domain in complex with unlabelled L-CICp (AXH:L-CIC-p ratio 1:1.2) recorded at 300 K on a Varian-Inova 800 MHz spectrometer. *Side chains* of glutamines and asparagines are indicated by a connecting line

Ataxin-1 is the protein associated with the human neurodegenerative spinocerebellar ataxia type 1 or SCA1 (de Chiara and Pastore 2011; Orr et al. 1993; Zoghbi and Orr 2009). The protein belongs to the family of the so-called polyglutamine proteins, all related to neurodegeneration, in which pathogenesis is associated with the expansion of a CAG repeat tract in the coding region of the gene. Although the disease is triggered by the elongation of the polyglutamine tract, which determines protein aggregation, it is now generally accepted that the protein context may modulate this process. Indeed, regions distant in sequence from the polyglutamines and interactions with molecular partners have been shown to play a role, either positive or negative, in modifying the pathology (de Chiara et al. 2009; Masino et al. 2011). One of such regions is the AXH domain of ataxin-1, which has been shown to contribute to aggregation of the expanded and non-expanded full length ataxin-1 in cells and to be sufficient to form fibres in vitro when isolated (de Chiara et al. 2005). The AXH domain of ataxin-1 is essential for the protein to function as a transcriptional co-repressor and mediates the majority of the interactions of ataxin-1 with cellular partners, mainly transcriptional regulators. The domain, which shares an oligonucleotide-binding fold and crystallizes as a dimer of asymmetric dimers, has been shown to be predominantly dimeric in solution and in equilibrium with monomers, tetramers and higher molecular weight species (de Chiara et al. 2013). The involvement of the domain in multiple association states and the presence of an extensive asymmetry at the interface between monomers are reflected in a very complex NMR spectrum which has hampered any possibility of achieving a complete resonances assignment of the free form of the domain (de Chiara et al. 2013). Interestingly, upon interaction with the ligand-peptide from CIC (L-CICp) the AXH domain forms a monomeric 1:1 complex with an NMR spectrum indicative of a single species (Fig. 1). Here we report the practically complete assignment of the complex between human ataxin-1 AXH and CIC.

## Materials and methods

### Protein expression and purification

The recombinant AXH domain of human ataxin-1 (residues A567–K689) was over-expressed in the *E. coli* host strain BL21 (DE3) using a kanamycin-resistant pETM30 vector with a TEV-cleavable N-terminal His6-GST tag. Isotopically ^15^N- and ^13^C/^15^N-labelled samples were expressed in minimal (M9) medium supplemented with ^15^N-ammonium sulphate and ^13^C-glucose as the sole sources of nitrogen and carbon respectively. Purification was performed using a Ni–NTA agarose column (Qiagen) followed by FPLC size exclusion chromatography as previously described (de Chiara et al. 2003). A synthetic unlabelled ligand peptide L-CICp spanning the sequence V34-Q48 of human CIC was purchased from Pepceuticals Limited (Nottingham-UK). AXH domain and L-CICp peptide concentrations were measured by UV absorbance at 280 nm using a calculated extension coefficient of 8,480 and 5,500 M^−1^ cm^−1^, respectively.

**Fig. 2:**
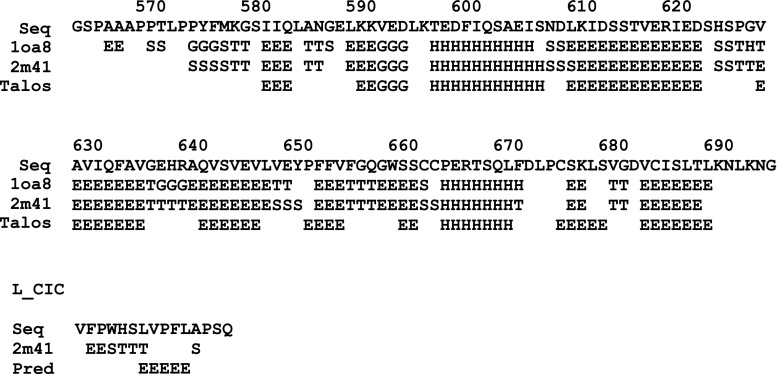
Comparison of the secondary structure as obtained from the chemical shifs of the complex and the X-ray structure of the free form (1ao8). The secondary structure of the resulting NMR structure determination of the complex is reported for comparison (2m41)

### NMR spectroscopy

NMR spectra for resonance assignments were acquired on samples containing ^15^N- or ^15^N,^13^C-labelled ATX1 AXH (0.5 mM) and unlabelled L-CICp (0.6 mM) (AXH:L-CICp molar ratio 1:1.2) in 20 mM Tris–HCl pH 6.85, 2 mM TCEP, 0.02 % NaN3, 8 % ^2^H_2_O. The spectra were recorded at 300 K using Varian Inova spectrometers operating at 600 and 800 MHz ^1^H frequency, the 800 MHz equipped with a triple resonance gradient Cold-Probe, and Bruker Avance spectrometers operating at 600 and 700 MHz ^1^H frequency, both equipped with triple resonance gradient CryoProbes.

All spectra were processed using NMRPipe/NMRDraw softwares (Delaglio et al. 1995) and analyzed using XEASY (Bartels et al. 1995).

### Resonance assignment and Deposition

Assignment of ^1^H, ^13^C and ^15^N of the AXH domain bound to unlabelled L-CICp was obtained as described below. Sequence specific ^1^HN, ^15^N, ^13^Cα, ^13^Cβ and ^13^C assignment for the AXH were obtained using HNCA, HN(CO)CA, HNCO and HNCACB (Muhandiram and Kay 1994) in combination with a 3D ^15^N-edited NOESY-HSQC experiment. Assignments of the ^1^H and ^13^C resonances of AXH aliphatic and aromatic side chains were obtained from HCCH-TOCSY experiments tuned for the two distinct regions of the spectrum (Sattler et al. 1999). Aromatic sequence specific side chain assignments were achieved using (Hβ)Cβ(CγCδ)Hδ and (Hβ)Cβ(CγCδ)Hε spectra supported by the identification of main-chain/side-chain NOEs from the ^13^C-edited NOESY-HSQC spectrum (100 ms mixing time). The proton resonances assignment of the CIC ligand-peptide was achieved using a combination of 2D [^15^N,^13^C]-F1/F2-filtered NOESY (mixing time 100 ms) and TOCSY (Otting and Wuthrich 1990). We assigned 100 % of the HN and N, 97 % of C and 96 % of H resonances of the backbone atoms and the side chains. No residues were completely unassigned.

Secondary structure predicted on the base of the chemical shifts using Talos+ (Shen et al. 2009) and CSI (version 2.0) (Wishart and Sykes 1994) for the protein and the peptide, respectively, have been compared with those of the complex and of the X-ray structure of the free form (Fig. 2). The ^1^H, ^13^C and ^15^N chemical shift of the ataxin-1 AXH domain and the ^1^H chemical shifts of the CIC ligand peptide have been deposited into the BioMagResBank database and are available under the BMRB accession number 18982.
